# Oncogenic effects of urotensin-II in cells lacking tuberous sclerosis complex-2

**DOI:** 10.18632/oncotarget.10748

**Published:** 2016-07-21

**Authors:** Alexander A. Goldberg, Kwang-Bo Joung, Asma Mansuri, Yujin Kang, Raquel Echavarria, Ljiljana Nikolajev, Yang Sun, Jane J. Yu, Stephane A. Laporte, Adel Schwertani, Arnold S. Kristof

**Affiliations:** ^1^ Meakins-Christie Laboratories, Translational Research in Respiratory Diseases Program, Montreal, Quebec, Canada; ^2^ Department of Critical Care Medicine, McGill University Health Centre Research Institute, Montreal, Quebec, Canada; ^3^ Department of Medicine, McGill University Health Centre, Montreal, Quebec, Canada; ^4^ Department of Pharmacology and Therapeutics, McGill University, Montreal, Quebec, Canada; ^5^ College of Medicine, University of Cincinnati, Cincinnati, Ohio, USA; ^6^ Division of Cardiology, Montreal General Hospital, Montreal, Quebec, Canada

**Keywords:** TSC2, mTOR, urotensin, lymphangioleiomyomatosis, angiomyolipoma

## Abstract

Lymphangioleiomyomatosis (LAM) is a destructive lung disease that can arise sporadically or in adults suffering from the tumor syndrome tuberous sclerosis complex (TSC). Microscopic tumors (‘LAM nodules’) in the lung interstitium arise from lymphatic invasion and metastasis. These consist of smooth muscle-like cells (LAM cells) that exhibit markers of neural crest differentiation and loss of the tumor suppressor protein ‘tuberous sclerosis complex-2’ (TSC2). Consistent with a neural phenotype, expression of the neuropeptide urotensin-II and its receptor was detected in LAM nodules. We hypothesized that loss of TSC2 sensitizes cells to the oncogenic effects of urotensin-II. TSC2-deficient Eker rat uterine leiomyoma ELT3 cells were stably transfected with empty vector or plasmid for the expression of TSC2. Urotensin-II increased cell viability and proliferation in TSC2-deficient cells, but not in TSC2-reconstituted cells. When exposed to urotensin-II, TSC2-deficient cells exhibited greater migration, anchorage-independent cell growth, and matrix invasion. The effects of urotensin-II on TSC2-deficient cells were blocked by the urotensin receptor antagonist SB657510, and accompanied by activation of Erk mitogen-activated protein kinase and focal adhesion kinase. Urotensin-II-induced proliferation and migration were reproduced in TSC2-deficient human angiomyolipoma cells, but not in those stably expressing TSC2. In a mouse xenograft model, SB657510 blocked the growth of established ELT3 tumors, reduced the number of circulating tumor cells, and attenuated the production of VEGF-D, a clinical biomarker of LAM. Urotensin receptor antagonists may be selective therapeutic agents for the treatment of LAM or other neural crest-derived neoplasms featuring loss of TSC2 or increased expression of the urotensin receptor.

## INTRODUCTION

Tuberous sclerosis complex (TSC) is a neurocutaneous tumor syndrome that occurs in approximately 1 in 6,000 live North American births [1, 2]. Clinical manifestations include tumors that consist of normally appearing tissue in unusual arrangements (*i.e.*, hamartomas). Tumors such as renal angiomyolipomas (AML) or lymphangioleiomyomatosis of the lung (LAM) cause significant morbidity and mortality. The abnormal cells (‘LAM cells’) in renal AMLs or pulmonary LAM nodules exhibit markers of neoplastic transformation, as well as smooth muscle and neural crest differentiation. LAM cells can invade the lymphatic circulation, metastasize, and survive in colonized tissue [3]. The molecular mechanisms that mediate the oncogenic phenotype are therapeutic targets in TSC and LAM [4].

TSC arises from heterozygous germline mutations in the tumor suppressor genes *TSC1* or *TSC2*, which are transmitted in autosomal dominant fashion. Second hit somatic mutations cause tissue-specific loss of the wild-type *TSC1* or *TSC2* allele (*i.e.*, loss of heterozygosity). *TSC1* and *TSC2* encode the proteins tuberin and hamartin; together they suppress mammalian target of rapamycin (mTOR), a central controller of cell growth, proliferation, and survival [5, 6]. The mTOR inhibitor rapamycin (Sirolimus, Rapamune™), or its analogues, inhibit the growth of TSC tumors [7–9]. However, their therapeutic efficacy can be limited by adverse effects related to blockade of mTOR in normal cells [10], as well as a paradoxical increase in cell survival resulting in the failure to eradicate tumors [11]. In addition, recent studies have revealed mechanisms of resistance to rapamycin, as well as mTOR-independent effects of TSC2 on cell differentiation and survival pathways [12, 13]. We investigated alternative mechanisms of neoplasia that might be specific to TSC2-deficient cells.

LAM cells express markers of neural crest differentiation, suggesting that neural crest-specific signaling molecules might represent therapeutic targets. We found high levels of urotensin-II (UII) and the urotensin receptor (UT) in tumor cells from lung biopsy tissue from patients with LAM [14]. Originally isolated from the neurosecretory system of Goby fish, the human form of UII was cloned, and encoded a peptide that bound an orphan G-protein-coupled receptor (*i.e.*, GPR14, UT) [15, 16]. The UT activates the G-protein Gαq/11, and the mobilization of intracellular calcium; via the same receptor, UII can also activate Gαi/o, the Erk MAPK pathway, and calcium release [17]. Moreover, UII promotes RhoA-dependent stress fiber formation, migration, and proliferation [18]. The mitogenic effect of UII has been less well studied, and is thought to occur primarily via activation of Erk MAPK [17]. UII binds its receptor with high affinity, and under normal conditions, the receptor is desensitized and inactive. Therefore, in contrast to most other G-protein-coupled receptors, elevated UT cell surface expression is required to accommodate sustained increases in extracellular UII. In agreement with low endogenous bioactivity of UII, the physiological phenotype of UT knockout mice is unremarkable [19]. The elevated UT expression observed in LAM nodules might indicate locally increased UII signaling related to their oncogenic behavior.

In addition to our observations in LAM, increased UII and UT levels were observed in several neoplasms of neural crest origin [20, 21]. A UT-dependent autocrine or paracrine mechanism for cell proliferation and survival was previously reported [22, 23]. We hypothesized that loss of TSC2 function imparts sensitivity to the mitogenic and pro-metastatic properties of UII in a mechanism that involves activation of the Erk MAPK pathway. Here we show that exposure of TSC2-deficient cells to UII increases viability, proliferation, migration, invasion, and colony formation in UT- and Erk-dependent fashion. Moreover, we show that pharmacological inhibition of UII signaling can inhibit the proliferation and growth of TSC-deficient cells and tumors in a mouse xenograft model.

## RESULTS

### Effect of UII on cell viability in TSC2-deficient cells

**Figure 1:**
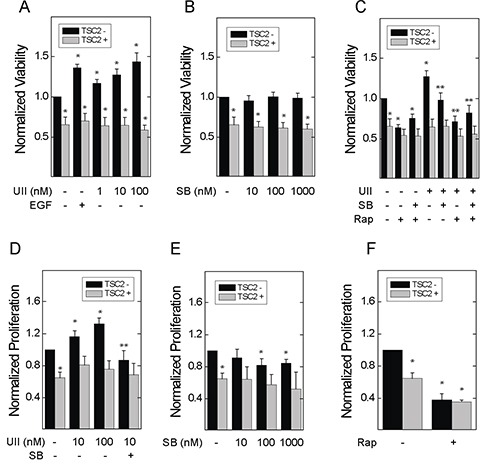
Effect of urotensin-II and the urotensin receptor inhibitor SB657510 on the viability and proliferation of TSC2-deficient cells In **A-C.**, empty vector transfected TSC2-deficient ELT3 (V3) or TSC2-reconstituted (T3) cells were incubated with serum-free medium for 4 h before addition of A, vehicle, 66 nM epidermal growth factor (EGF), or 1, 10, or 100 nM urotensin-II (UII), B, vehicle, 1, 10, or 100 nM SB657510 (SB), or C, vehicle, 10 nM UII, 10 nM SB, 50 nM rapamycin (Rap), or both for 48 h. After crystal violet staining, the corrected optical density (absorption 570 - 620 nm) was determined. Data are the means (± SE) of corrected optical density normalized to vehicle-treated V3 cell controls (mean 0.12 ± 0.02 O.D.) = 1 (n = 10 experiments each performed in triplicate). In **D-F**., V3 or T3 cells were incubated with serum-free medium for 24 h before addition of D, vehicle, 1, 10, or 100 nM UII, or 10 nM SB, E, vehicle, 1, 10, or 100 nM SB, or F, vehicle or 50 nM rapamycin (Rap), for 24 h. BrDU was assessed by ELISA. Data are the means (± SE) of corrected optical density (450-540 nm) normalized to vehicle-treated V3 cell controls (mean 0.4 ± 0.1 O.D.) = 1 (n = 7 experiments each performed in triplicate). * p < 0.05 *vs.* vehicle-treated V3 cell control, ** p < 0.05 *vs.* UII-treated V3 cells by Student's t-test. Heterogeneity was confirmed by two-way ANOVA in panels A-E (each p < 0.0001), with each p < 0.001 for TSC2- *vs.* TSC2+ cells.

To determine whether loss of TSC2 sensitizes cells to the oncogenic effects of UII, we used TSC2-deficient Eker rat uterine carcinoma (ELT3) cells stably transfected with empty vector (V3) or a plasmid for expression of TSC2 (T3) as previously described [24]. By crystal violet staining, viability was higher in V3 cells than that in T3 cells (Figure 1A). UII increased the viability of V3 cells, but not T3 cells, in concentration-dependent fashion, and the effect was similar to that of epidermal growth factor (EGF; Figure 1A), a known mitogen in TSC2-deficient cells [25]. Basal viability was unaffected by SB657510, a potent urotensin receptor (UT) antagonist (Figure 1B). However, SB657510 blocked the increase in cell viability observed in V3 cells treated with UII (Figure 1C). Unlike SB657510, the mTORC1 inhibitor rapamycin blocked basal and UII-induced cell viability in both V3 and T3 cells (Figure 1C). SB657510 did not further decrease viability in cells exposed to rapamycin. We next assessed cell proliferation by BrDU incorporation assay as a potential mechanism for increased cell viability in TSC2-deficient cells. UII increased proliferation in concentration-dependent fashion in V3, but not T3 cells, and SB657510 blocked UII-induced proliferation (Figure 1D). At higher concentrations (100 or 1000 nM), SB657510 alone inhibited proliferation in V3 cells, but not in T3 cells (Figure 1E). Rapamycin blocked proliferation in both TSC2-deficient and reconstituted cells (Figure 1F). These results indicate that UII increases viability and cell proliferation specifically in TSC2-deficient cells, and in a mechanism that can be blocked by the UT antagonist SB657510.

### Effect of UII on cell migration and detachment in TSC2-deficient cells

**Figure 2:**
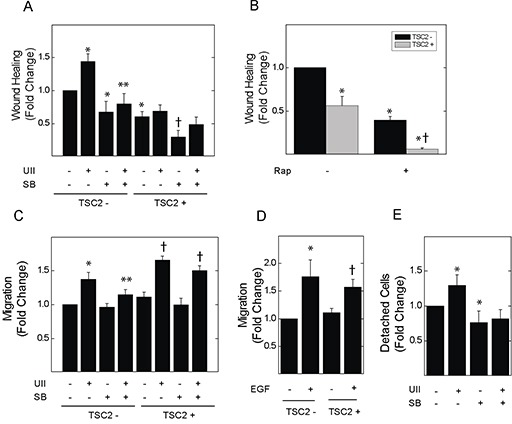
Effect of urotensin-II and the urotensin receptor inhibitor SB657510 on cell migration or detachment of TSC2-deficient cells In panels **A** and **B.**, V3 or T3 cells were incubated with serum-free medium for 4 h before initiation of the wound-healing assay, and the subsequent addition of A, vehicle, 10 nM UII, 10 nM SB, or both, or B, vehicle or 50 nM rapamycin (Rap) for 8 h. The percentage decrease in cell-free area (*i.e.*, migration, wound healing) was determined by imaging as described in the Methods section. Data in A and B are the means (± SE) of percent wound healing normalized to vehicle-treated V3 cells (mean 13.3 ± 3.2%) = 1 (n = 5 experiments each performed in duplicate). In panels **C** and **D.**, V3 or T3 cells were seeded in Transwell culture chambers (10,000 cells per well) and incubated with serum-free medium for 24 h before addition to the lower chamber of C, vehicle, 10 nM UII, 10 nM SB657510, or both, or D, vehicle or 66 nM EGF. Data are the means (± SE) of cell number per 10 high-powered fields normalized to vehicle-treated V3 controls (mean 28.4 ± 2.6 cells) = 1 (n = 5 experiments each performed in duplicate). **E.**, 250,000 V3 cells were seeded in 100 mm culture dishes, and cultured overnight before exposure to serum-free medium for 4 h followed by vehicle, 10 nM UII, 10 nM SB657510, or both for 24 h. Detached cells in culture supernatants were collected and counted by hemocytometry. Data are the means (± SE) of detached cell number per 100 mm plate normalized to vehicle-treated V3 cells (mean 26,175 ± 4,385 cells) = 1 (n = 5 experiments each performed in duplicate). * p< 0.05 *vs.* vehicle-treated V3 cells, ** p < 0.05 *vs.* UII-treated V3 cells, † p < 0.05 *vs.* vehicle-treated T3 cells by Student's t-test. Heterogeneity was confirmed by two-way ANOVA in panels A-C (each p < 0.0001), with each p<0.001 for TSC2- *vs.* TSC2+ cells. For panel D, p < 0.008 by two-way ANOVA, and p not significant for TSC2- *vs.* TSC2+ cells. For Panel E, p = 0.04 by one-way ANOVA.

UII can stimulate the migration of macrophages [26], and can activate Erk-dependent cytoskeletal rearrangement [17]. Moreover, Erk plays a central role in focal adhesion turnover during cell migration [27]. In *in vitro* wound healing assays, incubation with UII for 8 h induced the migration of TSC2-deficient V3 cells; basal and UII-induced migration were blocked by SB657510 (Figure 2A). UII failed to induce migration in TSC2-reconstituted T3 cells. As was the case for cell viability and proliferation, baseline migration was elevated in TSC2-deficient V3 cells compared to that in T3 cells. Rapamycin reduced migration in both V3 and T3 cells (Figure 2B). To assess chemotaxis, V3 or T3 cells were seeded in the upper chamber of a dual-chamber culture system with separating membranes perforated by 8 μm pores, before addition of vehicle or UII without or with SB657510 to the lower chamber for 24 h. UII increased the number of TSC2-deficient V3 cells migrating to the lower chamber, and this was blocked by SB657510 (Figure 2C). SB657510 failed to block UII-enhanced chemotaxis in cells expressing TSC2. The levels of UII-induced chemotaxis were similar to those induced by EGF (66 nM) (Figure 2D). Although UII-induced migration appeared greater in T3 cells, this effect may have been due to greater detachment of V3 cells from the upper chamber of the Transwell insert during the 24 h incubation period [28]. In agreement, the number of detached V3 cells isolated from the culture supernatants was greater than that observed in T3 cell cultures; UII enhanced the detachment of V3 cells, and this was blocked by SB657510 (Figure 2E). Thus, UII-induced migration and chemotaxis are blocked by SB657510 in TSC2-deficient cells.

### UII stimulates the anchorage-independent cell growth, invasion and colony formation of TSC2-deficient cells

**Figure 3:**
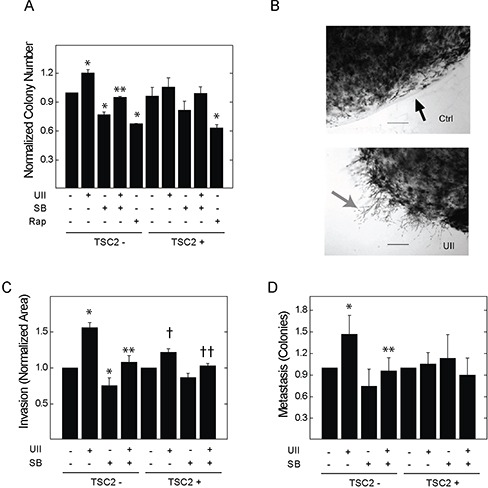
Effect of urotensin-II and the urotensin receptor inhibitor SB657510 on anchorage-independent cell growth and fibrin matrix invasion in TSC2-deficient cells **A.**, V3 or T3 cells (10,000 cells) were cultured on soft agar as described in the Methods section. Cells were exposed to vehicle, 10 nM UII, 10 nM SB, or both, or 50 nM rapamycin for 14 d before staining with crystal violet, microscopic imaging, and colony counting. Data are the means (± SE) of the average number of colonies per 10 high-powered fields normalized to vehicle-treated V3 cells (mean 16.8 ± 3.5 colonies) = 1 (n = 3 experiments each performed in duplicate). In **B-D.**, V3 or T3 cells (50,000 cells) were embedded in 200 μl collagen plugs and sandwiched between two layers of fibrin before exposure to vehicle, 10 nM UII, 10 nM SB657510, or both. B, After staining with methylene blue and imaging (magnification 40x, scale bar = 2.5 mm), the area representing cell invasion (grey arrow) beyond the perimeter (black arrow) of the collagen plug was measured, and colonies that were distant from the perimeter were counted. C, Data for local invasion are the means (± SE) of the average invasion area divided by perimeter length for 5 high-powered fields per 100 mm plate normalized to vehicle-treated V3 (mean 40.9 ± 3.5) = 1 or T3 cells (mean 111.3 ± 15.8) = 1 (n = 5 experiments each performed in duplicate). D, Data for distant colony number (*i.e.*, metastasis) are the means (± SE) of the total colony number normalized to vehicle-treated V3 (mean 17.6 ± 7.3 colonies) = 1or T3 cells (mean 14.5 ± 4.8 colonies) = 1 (n = 5 experiments each performed in duplicate). * p < 0.05 *vs.* vehicle-treated V3 control, ** p < 0.05 *vs.* UII-treated V3 cells, † p < 0.05 *vs.*vehicle-treated T3 cells, †† p < 0.05 *vs.* UII-treated T3 cells by Student's t-test. Heterogeneity was confirmed by two-way ANOVA in panels A-C (p < 0.0001), with p<0.001 for drug effect.

TSC2-deficient V3 or reconstituted T3 cells were cultured in soft agar for one week. Incubation with UII significantly increased colony growth in V3, but not T3, cells (Figure 3A). Basal and UII-stimulated colony formation were blocked by SB657510 in V3 cells. Rapamycin blocked colony formation to a similar extent in V3 and T3 cells. To determine whether UII stimulates the invasion of fibrin matrix by TSC2-deficient cells, we embedded V3 or T3 cells in a collagen plug, which was then cultured in a fibrin matrix as previously described [29]. Exposure to UII enhanced invasion of V3 cells in fibrin matrix to a greater extent than that observed in T3 cells (Figure 3B, 3C); SB657510 blocked both basal and UII-stimulated invasion. In the same assay, we observed the formation of colonies distant from the collagen plug as an indicator of *in vitro* ‘metastasis’ [29]. Similar to invasion, UII augmented distant colony formation, and this was blocked by SB657510 (Figure 3D). These results indicate that UII can promote the survival and matrix invasion properties of TSC2-deficient cells.

### Erk MAPK and Fak mediate the effect of UII on proliferation and migration

**Figure 4:**
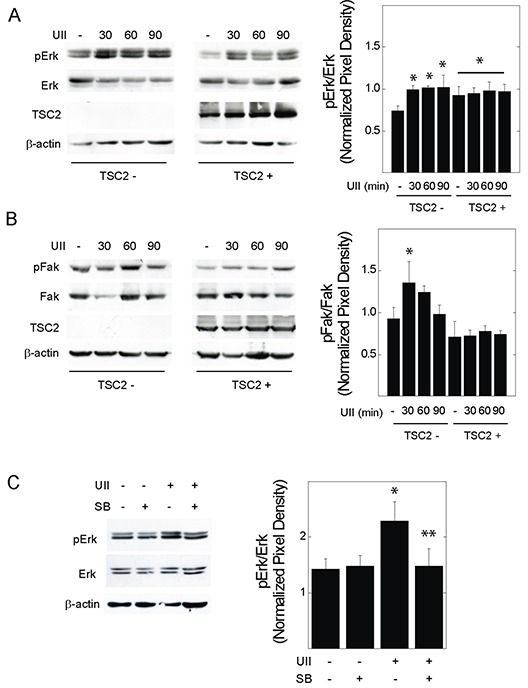
Effect of urotensin-II on the phosphorylation of Erk or Fak in TSC2-deficient cells V3 or T3 cells were incubated in serum-free medium overnight before exposure to vehicle or 10 nM UII for 0, 30, 60, or 90 min, and preparation of whole cell lysates. Proteins indicating **A.**, Erk or **B.**, Fak activity were detected by Western blot. In **C.**, V3 cells were exposed to vehicle, UII, SB, or both for 30 min before Western blot analysis. Shown to the right of each blot are summarized densitometry data representing the means (± SE) of pErk or pFak divided by total Erk or Fak levels (n = 3-5 experiments). * p < 0.05 *vs.* V3 control, **p < 0.05 *vs*. UII-treated V3 cells by Student's t-test.

**Figure 5:**
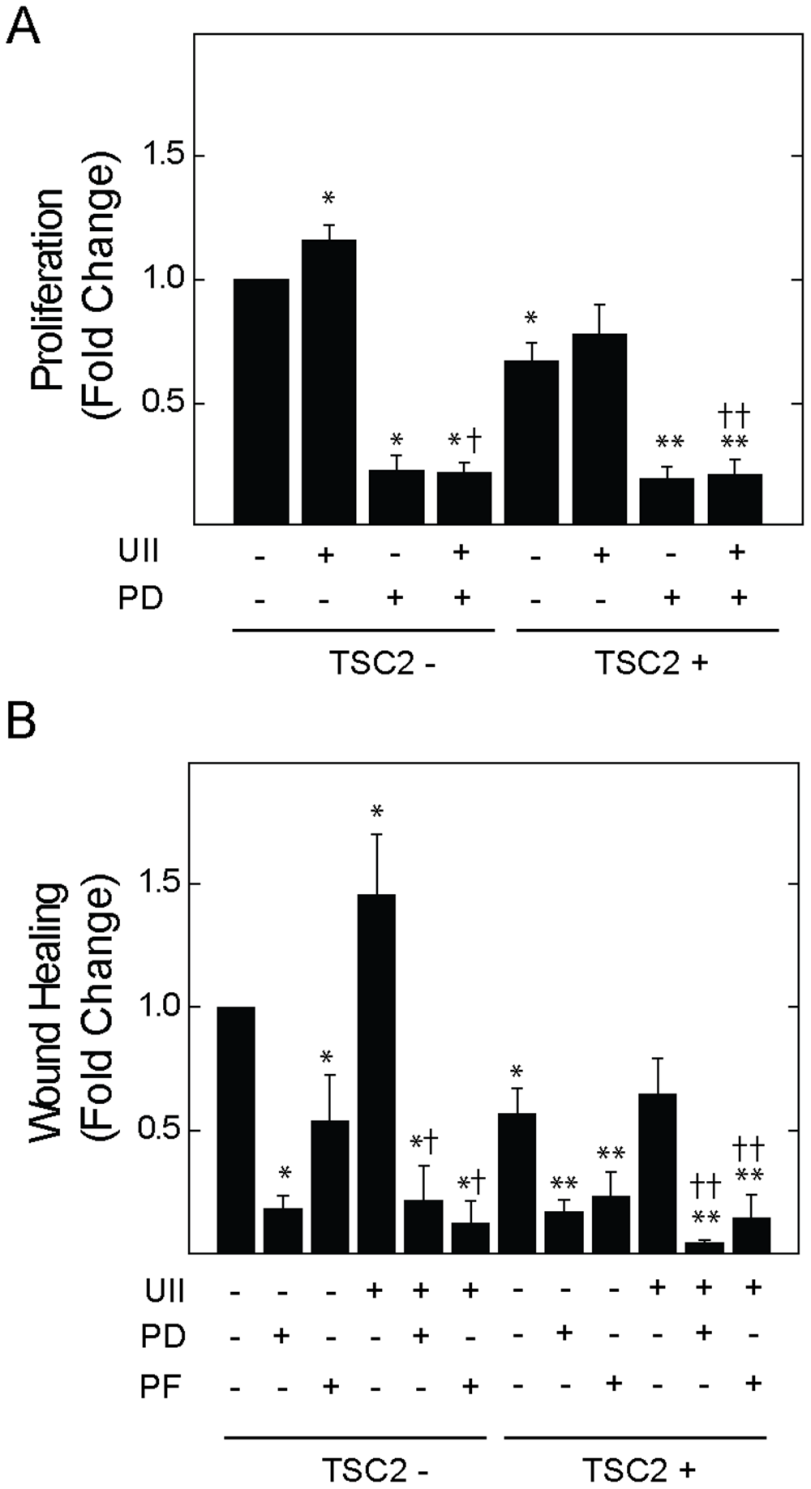
Effect of Erk MAPK kinase or Fak inhibition on UII-induced proliferation or migration of TSC2-deficient cells V3 or T3 cells were incubated in serum-free medium for 4 h before exposure to **A.**, vehicle, 10 nM UII, 2 μM PD184352 (PD), or both for 24 h, and measurement of proliferation, or **B.**, vehicle, 2 μM PD184352 (PD), or 2 μM PF-223 (PF) alone or in the presence of 10 nM UII, before assessment of cell migration by wound healing assay. Data are the means (± SE) of BrDU incorporation (A) or percent wound healing (B) normalized to untreated V3 control (mean 0.4 ± 0.1 O.D. or 17.0 ± 5.0%) = 1 (n = 4-6 experiments each performed in triplicate). * p < 0.05 *vs.* vehicle-treated V3 control, ** p < 0.05 *vs.* vehicle-treated T3 cells, † p < 0.05 *vs.* UII-treated V3 cells, †† p < 0.05 *vs.* UII-treated T3 cells by Student's t-test.

Erk MAPK is an effector of UII signaling, and regulates the turnover of focal adhesion during cell migration and invasion [27]. V3 or T3 cells were exposed to UII for 0 to 90 min, before detecting phosphorylation of Erk1 and 2 at Thr202 and Tyr204. UII increased the phosphorylation of Erk in time-dependent fashion (Figure 4A). The induction of Erk phosphorylation by UII was greater in TSC2-deficient V3 cells than that observed in TSC2-reconstited T3 cells. When normalized to total Fak levels, phosphorylation of Fak at Y397 was increased by UII in V3 cells, but not in TSC2-reconstituted T3 cells (Figure 4B). Consistent with increased Erk activity and focal adhesion turnover [30], there was a transient reduction in total Fak levels after exposure to UII in V3 cells, but not in T3 cells (Figure 4B). The increase in Erk phosphorylation was reversed by the urotensin receptor blocker SB657510 (Figure 4C). The Erk inhibitor PD184352 decreased basal and UII-induced proliferation by BrDU incorporation assay (Figure 5A). Similarly, PD184352, as well as the Fak inhibitor PF-228, potently inhibited basal and UII-induced migration by scratch assay (Figure 5B). In contrast to SB657510, the Erk and Fak inhibitors blocked proliferation and migration in TSC2-reconstituted cells, suggesting that TSC2-deficiency modifies proliferation and migration by a mechanism upstream of Erk during engagement of the urotensin receptor.

### The urotensin receptor is required for basal and UII-induced migration in TSC2-deficient cells derived from a patient with LAM

**Figure 6:**
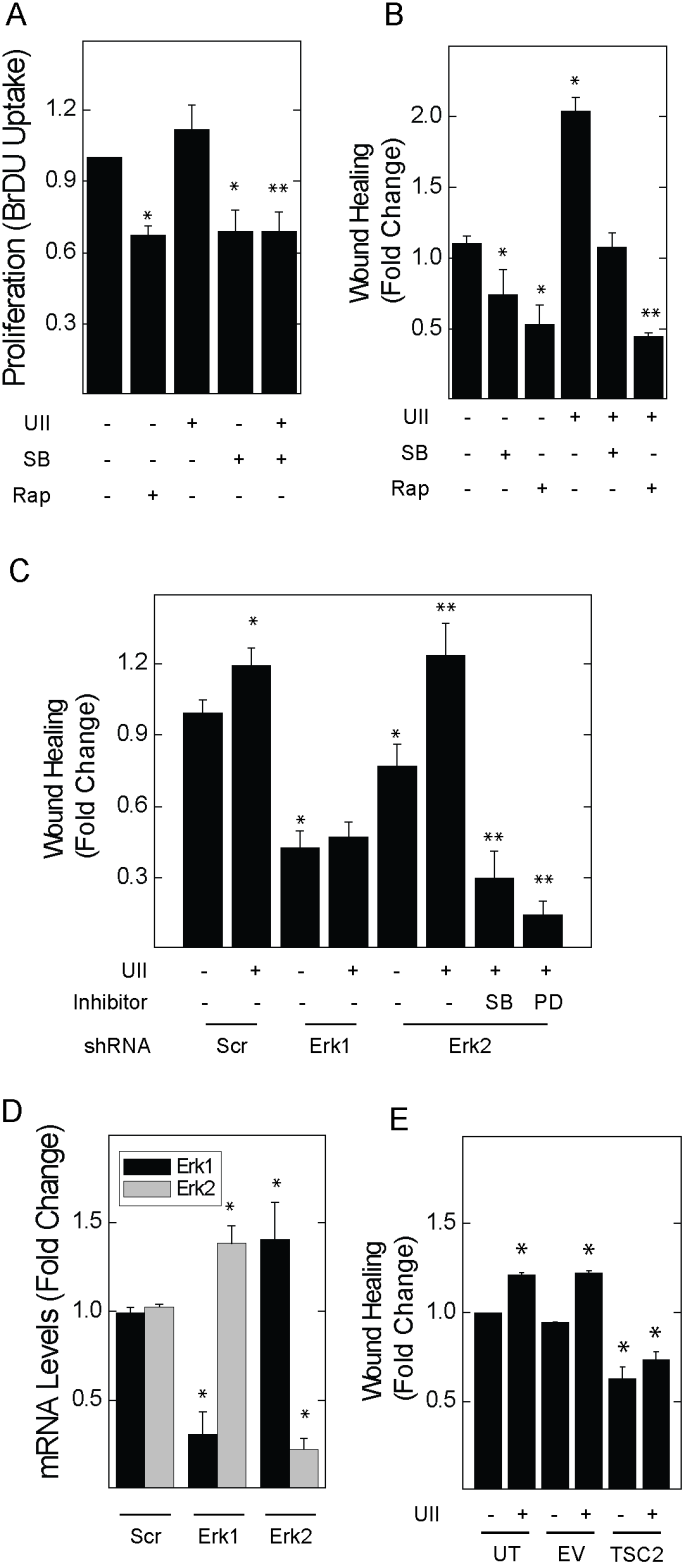
Inhibitors of urotensin signaling block UII-induced proliferation and migration in human angiomyolipoma cells **A.**, 621-101 cells were exposed to vehicle, 10 nM UII, 10 nM SB657510, or 50 nM rapamycin for 24 h, before measurement of BrDU incorporation. **B.**, Cell monolayers were wounded before addition of vehicle, 50 nM rapamycin, 10 nM UII, 10 nM SB657510, or both for 8 h. In **C.**, scratch assays were performed in cells transduced with lentiral vectors for the expression of control shRNA (Scr) or that targeting Erk1 or Erk2 for 48 h before exposure to vehicle or 10 nM UII in the absence or presence of 10 nM SB657510 or 2 μM PD184352. **D.**, 621-101 cells were transduced with control (Scr), α-Erk1, or α-Erk2 shRNA before determination of Erk1 and Erk2 mRNA levels by qPCR. **E.**, 621 cells stably transfected with empty vector or that encoding the expression of TSC2 were wounded before exposure to vehicle or 10 nM UII for 8 h. In A and B, data are the means (± SE) of BrDU incorporation or percent wound healing normalized to vehicle-treated 621 cells (0.35 ± 0.01 O.D.) = 1 (n = 4 experiments each performed in duplicate); * p< 0.05 *vs.* control, ** p < 0.05 *vs.* UII-treated cells by Student's t-test. In C-E, data are the means (± SE) of percent wound healing or mRNA levels normalized to vehicle-treated untransfected (18.5 ± 2.1%) = 1 or scrambled shRNA-transduced controls (16.1 ± 1.9%) = 1 (n=3 experiments each performed in triplicate); * p< 0.05 *vs.* scrambled or untransfected control, **p < 0.05 *vs.* vehicle treated Erk2-depleted cells by Student's t-test.

621-101 cells are TSC2-deficient cells derived from the renal angiomyolipoma of a patient with LAM [31, 32]. UII led to a small increase in the proliferation of 621-101 cells (Figure 6A); similar to rapamycin, SB657510 decreased the basal proliferation of 621-101 cells. We focused on migration as a dominant oncogenic phenotype in TSC2-deficient cells exposed to UII. UII increased the migration of 621 cells by scratch assay; SB657510 blocked UII-induced migration (Figure 6B). The inhibitory effect of SB657510 was similar to that of rapamycin. Depletion of Erk1 by shRNA blocked basal and UII-induced cell migration in 621 cells (Figure 6C). Depletion of Erk2 led to a paradoxical increase in UII-induced migration (Figure 6C), perhaps due to a compensatory increase in Erk1 expression (Figure 6D). Administration of SB657510 or PD184352 reversed the increase in migration produced by depletion of Erk2 (Figure 6C). Stable expression of TSC2 in 621 cells (*i.e.*, 621-103 cells), which was previously shown to restore TSC2 expression and reduce mTORC1 activity [32], attenuated the cell migration observed in empty vector-transfected controls (*i.e.*, 621-102 cells), and abolished the migratory response to UII (Figure 6E). Thus, UII and the urotensin receptor mediate a pro-oncogenic phenotype in TSC2-deficient human angiomyolipoma (621) cells that requires Erk1.

### SB657510 attenuates the growth and invasion of TSC2-deficient tumors in a mouse xenograft model

**Figure 7:**
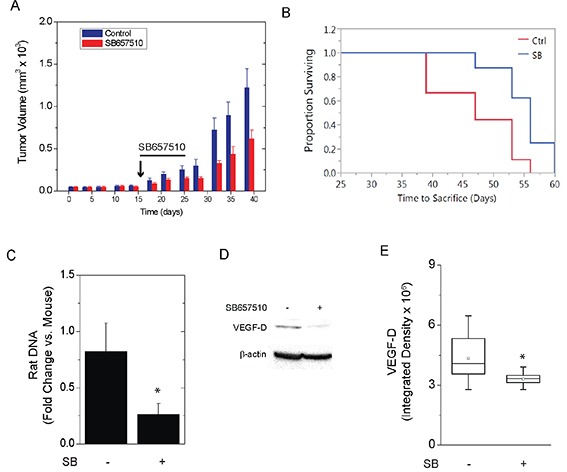
SB657510 inhibits growth of TSC2-deficient tumors *in vivo* ELT3-luc cells were injected subcutaneously in the flanks of SCID mice. On day 17, mice received vehicle (0.1% methylcellulose) or SB657510, 50 mg/kg, daily for 9 d. **A.**, Mean tumor volume normalized to vehicle control on Day 0 (47.6 ± 7.2 mm^3^) = 1 (p = 0.03 by repeated measures ANOVA, p = 0.03 SB657510 *vs.* vehicle control, n=20) or **B.**, survival time from detection of palpable tumor to sacrifice for animals in panel A (p = 0.01 by Log rank test) are shown. **C.**, At necropsy, circulating blood cells were separated by centrifugation. DNA was isolated for measurement of mouse short interspersed nuclear elements (mSINEs) or rat long interspersed nuclear elements (rLINEs) by qPCR. The fold changes in rLINE relative to mSINE DNA copies (mean ± SEM, n=6) are shown (* p = 0.04 rLINE *vs.* mSINE DNA). **D.**, Western blot analysis for VEGF-D with **E.**, box plots showing quantification of band density (p < 0.05 by Wilcoxon rank sum test, n=16).

ELT3-luc cells were injected subcutaneously into the flanks of SCID mice. Upon palpation of established tumors, vehicle (0.1% methylcellulose) or SB657510 (50 mg/kg) were administered by oral gavage for 9 days. The growth of ELT3 cell tumors was significantly attenuated by the administration of SB657510 (Figure 7A). Consistent with reduced tumor growth, the probability of survival from first detection of tumor was increased by the administration of SB657510 (Figure 7B). Moreover, the number of circulating rat cells at necropsy, as measured by qPCR detection of rat long interspersed nuclear elements (rLINEs), was significantly lowered by the administration of SB657510, suggesting that invasion of tumor cells into the vascular space was reduced (Figure 7C). Finally, SB657510 significantly reduced the levels of VEGF-D, a known biomarker of LAM, in tumor lysates (Figure 7D, 7E).

## DISCUSSION

Inherited loss of the TSC2 is associated with the development of hamartomatous tumors in multiple organs [1]. Despite the relative benign nature of these tumors, emerging evidence indicates that TSC2-deficient cells exhibit characteristics of low-grade neoplasia, including invasion of extracellular matrix and metastasis to distant organs [33]. In addition, the expression of multiple neural crest cell markers supports a probable neuro-endocrine role in the pathogenesis of TSC and LAM [24, 34]. The mechanisms by which TSC2-deficiency renders cells susceptible to neoplastic behavior are therefore of major interest. Here, we show that the neuropeptide urotensin-II (UII) increases viability, proliferation, migration, and invasion in TSC2-deficient cells, but not their TSC2-reconstituted counterparts. The magnitude of the effect of UII was similar to that observed for epidermal growth factor, a known inducer of growth and survival in TSC2-deficient cells [35]. The findings applied to two established *in vitro* models of TSC2-deficiency: Eker rat uterine leiomyoma cells and human angiomyolipoma 621 cells. Finally, we show that the urotensin receptor antagonist SB657510 can inhibit the growth and blood stream invasion of TSC2-deficient ELT3 cells in a mouse xenograft model, as well as the expression of VEGF-D, a known biomarker related to LAM.

Our results demonstrate a robust effect of urotensin receptor on parameters related to tumor metastasis and survival. In agreement, the effects of UII on migration in TSC2-deficient cells required the induction of proliferative and cytokinetic signaling pathways commonly increased in cancer (*i.e.*, Erk MAPK, Fak). Although the urotensin receptor, and its role in cell proliferation and migration in the cardiovascular system, has been studied [17, 36], less is known regarding the molecular mechanisms that promote urotensin signaling in neoplastic cells. In fact, the UII receptor (UT, GPR14), can activate the G-protein Gαq/11, and the mobilization of intracellular calcium via phospholipase C and inositol triphosphate [17]. The urotensin receptor can also activate Gαi/o and Erk MAPK. UII causes RhoA-dependent stress fiber formation, migration, and proliferation via Gαq/11, although Gα12/13 may also be involved. Both RhoA and Erk are mitogenic, and can regulate focal adhesion turnover [37, 38]. Enhanced Rho and Rac signaling have been implicated in the pathogenesis of TSC and LAM [10, 26, 39].

In addition to tumor growth, SB657510 reduced the number of circulating tumor cells, suggesting its blockade of migration and vascular invasion *in vivo* (Figure 7). Interestingly, certain phenotypes (*e.g.*, fibrin invasion, chemotaxis) appeared more prominent in ELT3 cells expressing TSC2. This apparent discrepancy was also observed in other studies [38]. For instance, over-expression of TSC2 in epithelial cells was associated with increased migration, RhoA activation, and phosphorylation of Fak [24]. In our study, perhaps due to lower total Erk levels in TSC2-reconstituted ELT3 cells [40], the baseline ratio of phosphorylated to total Erk was augmented in TSC2-expressing cells (Figure 4). Furthermore, basal invasion of fibrin matrices was more pronounced in ELT3 cells expressing TSC2 (Figure 3), possibly due to increased adhesiveness of TSC2-expressing cells [24], or reduced adhesiveness of TSC2-deficient cells (Figure 2E) [28]. In agreement with these findings, TSC tumors exhibit a relatively benign phenotype, suggesting that loss of TSC2 creates a balance between anti- and pro-oncogenic mechanisms. For example, excessive mTORC1 activity can suppress growth factor-induced cell survival by a negative feedback mechanism involving the attenuation of Akt [11]. Moreover, cells in TSC or LAM tumors are not completely autonomous, and require exogenous factors (*e.g.*, estrogen) for invasion and metastasis [41]. Nonetheless, our results demonstrate that loss of TSC2 renders cells more sensitive to the oncogenic effects of UII and to inhibition by the urotensin receptor blocker SB657510.

In agreement with our results, others have demonstrated a fundamental role for Erk and Fak in the invasive phenotype of neoplastic cells (reviewed in [42]). Although increased Fak activity in cancer cells is thought to drive Erk activity and proliferation, a number of studies have indicated that Erk can directly regulate focal adhesion turnover, at least in part by phosphorylation of Fak [30, 43]. UII did not alter the phosphorylation of Fak in cultured smooth muscle cells [44], suggesting that the loss of TSC2 in rat ELT3 cells might lower the threshold for agonist-induced migration and invasion. Consistent with a mechanism upstream of Fak, we observed that Erk1, but not Erk2, was required for UII-induced migration of human angiomyolipoma (621-101) cells (Figure 6C). Moreover, inhibition of migration by the UII receptor blocker SB657510, or by the Erk signaling inhibitor PD184352, was greater in cells with enhanced Erk1 expression (*i.e.*, Erk2-depleted cells; Figure 6C). In contrast to UII, the effects of estrogen on migration and epithelial-mesenchymal transition in TSC2-deficient cells required Erk2 [45]. The relative role of Erk isoforms in TSC2-deficient cells may depend on the agonist or signal transduction mechanism.

Together, the findings, along with our previous study demonstrating elevated levels of UII and its receptor in the tumor nodules of patients with pulmonary LAM (LAM nodules) [14], suggest a prominent and specific role for UII signaling in LAM, and possibly other neoplastic conditions featuring increased urotensin signaling. Although rapamycin has been used to delay the clinical progression of TSC and LAM [8, 9, 37], its administration has been associated with adverse metabolic and inflammatory effects [10]. Rapamycin's target, mTOR, is a ubiquitous protein that regulates numerous cellular processes, and our data show that rapamycin affects proliferation and viability in cells irrespective of the presence of TSC2 (Figure 1). In the current study, urotensin signaling was only enhanced in TSC2-deficient cells, suggesting that urotensin receptor antagonists might specifically target the growth and metastasis of TSC tumors without fewer side effects. Moreover, augmented urotensin signaling in TSC or LAM tumors is consistent with the expression of other immature neural crest markers (*e.g.*, gp100, tyrosinase), and its role in other neoplasias of neural or endocrine lineage [39, 46, 47]. Encouragingly, SB657510 reduced tumor growth, vascular invasion, and tumor VEGF-D levels in mice without apparent toxicity (Figure 7). Urotensin receptor antagonists have been safely administered to human subjects in clinical trials [48], and they represent potential therapeutic avenues to prevent or slow the progression of LAM and TSC.

## MATERIALS AND METHODS

### Cell culture and reagents

Tuberin-deficient Eker rat uterine leiomyoma (ELT3) cells [49] were further studied by Astrinidis A. *et al.*, who developed the stably transfected ELT3 V3 (empty vector) and ELT3 T3 (vector for mammalian expression of TSC2) cell lines [24]. 621-101 human angiomyolipoma cells were derived and propagated by transformation of kidney angiomyolipomas excised from a patient with LAM (Dr. E. Henske, Harvard University) [31]. 621-102 and 621-103 cells are 621-101 cells stably-transfected with empty vector and that for the constitutive expression of TSC2, respectively [32]. For RNAi-mediated depletion, cells were incubated with shRNAs designed to target Erk1 or Erk2 (gifts from P. Roux, Université de Montréal), or scrambled control (Addgene), as described previously [50]. All cell lines were cultured in DMEM supplemented with or without 10% fetal bovine serum in the presence of penicillin, 100U/ml, and streptomycin, 100 μg/ml. Reagents used were urotensin-II (Tocris), urotensin receptor inhibitor SB657510 (Tocris), MEK inhibitor PD184352 (LC Laboratories), Fak inhibitor PF-228 (Tocris), mTORC1 inhibitor rapamycin (Millipore), and epidermal growth factor (Sigma Aldrich).

### Cell viability, proliferation, and counting

Cell viability was assessed by incubating washed cells with 0.2% crystal violet for 15 min. Stained cells were washed with PBS and solubilized in 1% sodium dodecyl sulfate (SDS) solution for 15 min. The optical density (OD) of each sample was determined by correcting the optical density at 570 nm by that at 620 nm. DNA synthesis as an index of cell proliferation was assessed by BrdU Assay as per the manufacturer's recommendations (Roche). In cell counting experiments, 250,000 cells were seeded and grown for 24 h in 10 cm dishes before initiation of experiments. Detached cells were collected from the media supernatants by low-speed centrifugation and counted by hemocytometry.

### Cell migration and chemotaxis

For wound healing (‘scratch’) assay, a cross-shaped defect was created in cell monolayers before incubation with growth factors or pharmacological inhibitors as indicated. Images were acquired (Olympus IX70 microscope) upon creation of the scratch, and 8 h later. Using image analysis software (ImagePro Plus), the change in area of the defect over 8 h was measured. Results are the area of the defect before incubation with reagents minus the area at 8 h, all divided by the pre-incubation area. Chemotaxis was evaluated by Transwell chamber migration assay. 10,000 cells were suspended in serum-free DMEM medium and added to the upper compartment of a 6.5 mm diameter Transwell insert with 8 μm pores (Corning). Serum-free medium without or with UII or SB657510 was added to the lower chamber, and cells in the upper chamber were allowed to migrate for 24 h. The cells that remained on the upper surface of the membrane were removed with a cotton swab before staining cells that migrated to the lower surface with crystal violet, and counting by microscopy.

### Anchorage-dependent cell growth and matrix invasion

1 ml of pre-warmed (37°C) 2× Iscove's modification of Dulbecco's medium (IMDM) (20% FBS, 4 mM L-Glu, 2×NEAA, 0.6% sodium bicarbonate, 2% sodium pyruvate, 200 U/mL penicillin/streptomycin; Invitrogen) was added to 1.5 ml pre-warmed (56°C) 0.8% Bacto Agar and 0.5 ml cell suspension, and then seeded over a 0.6% agar/IMDM pre-layer (3 ml) in a 35 mm dish. Semisolid 0.6% feeder layers (3 ml) were overlaid atop the solid cell layers. After 4 d, colony numbers were determined by visual counting using a microscope. For matrix invasion assay, cells were embedded in collagen gel (50,000 cells per 200 μl of collagen solution) in 96-well plates, as described previously [29], sandwiched between fibrin gel layers, and placed in 24-well plates before incubation for 4 days in 1 ml medium supplemented without or with growth factors or pharmacological inhibitors as indicated. Media, supplemented with the anti-fibrinolytic agent aprotinin (100 KIU/ml, Bioshop), were replaced every other day. Cells were stained using 0.2% methylene blue in 50% methanol, and representative images of cells at the perimeters of the plugs, as well as colonies distant from the plugs, were acquired (Olympus XL60 microscope, 40x). Area of invasion was defined as that including cells invading beyond the perimeter of the collagen plug and was measured using image analysis software (ImagePro Plus).

### Protein and mRNA detection

Whole cell lysates were generated after washing cells once with cold PBS, and incubating for 15 min on ice in lysis buffer (1% NP-40, 50 mM Tris, 5 mM EDTA, 10 mM β-glycerophosphate, aprotinin, 10 μg/ml, leupeptin, 10 μg/ml, 1 mM PMSF, 50 mM NaF, 100 μM sodium orthovanadate). After freezing and thawing, particulate-free cytoplasmic lysates were generated. Equal amounts of proteins were probed for levels of phospo-Erk 1/2 MAPK (T202/Y204), total Erk 1/2 MAPK, phospho-p70S6K, total p70S6K, phospho-FAK (Y397), total FAK, TSC2, and β-actin (Cell Signaling), as well as VEGF-D (Abcam) by Western blot analysis. Gel images were acquired using Alpha Imager (Alpha Inotech Corp) and analyzed with Alpha Ease FC software (version 4.1.0). Band density was measured using the spot density and auto-background functions. For quantitative real-time PCR, RNA was extracted, and cDNA was generated by reverse transcription from 2 μg RNA. Sybr green-based real-time PCR was performed using 1 μl cDNA and Sybr green Universal PCR master mix (ABI). PCR using template-specific primers as indicated in Supplementary Table S1 was performed (ABI 7500 Real Time PCR System). Results are the fold induction in mRNA levels as determined by the ΔΔCt method [51].

### *In vivo* tumor xenograft growth

Animal protocols were approved by the animal care committee at McGill University (Montreal, Canada). TSC2-deficient ELT3-luc cells were injected subcutaneously in the flanks of 8-12 week old female SCID hairless congenic mice (Charles River). Palpable tumors were first detected 8 d post-injection. On day 17, mice received vehicle (0.1% methylcellulose) or SB657510, 5 mg/kg, by oral gavage daily for 9 d. Mice were killed via carbon dioxide inhalation when tumor volume exceeded 2000 mm^3^. Tumors were harvested for protein and mRNA analysis. Blood was taken from mice at necropsy, and blood cells were separated by differential centrifugation before isolation of DNA for measurement of mouse short interspersed nuclear elements (mSINEs) or rat long interspersed nuclear elements (rLINEs) by qPCR. Primer sequences are indicated in Supplementary Table S1.

### Statistical analysis

Statistically significant differences (p< 0.05) between means were calculated for *in vitro* experiments by Student t-test or analysis of variance (ANOVA). For *in vivo* experiments, Repeated Measures ANOVA was performed to assess for heterogeneity between the treatment groups in mice over time. For survival analysis, log rank test was performed to assess for significant differences in survival between the treatment groups. Statistical analyses were performed using JMP 11 software.

## SUPPLEMENTARY TABLE


