# Supplementary material for: Oncogenic effects of urotensin-II in cells lacking tuberous sclerosis complex-2
Source: Oncotarget. 2016 Jul 21;7(38):61152–65. doi: 10.18632/oncotarget.10748 (PMC5308642; doi:10.18632/oncotarget.10748)
Supplement: Supplementary file 1 [file oncotarget-07-61152-s001.pdf]

Oncogenic effects of urotensin-II in cells lacking tuberous sclerosis complex-2

SUPPLEMENTARY TABLE

Supplementary Table S1: Oligonucleotide primers (5'-3') for Sybr Green-based Real-time PCR

Target	Species	Forward	Reverse	Amplicon (bp)
Erk1	Human	GATCAGCCCCTTCGAACATCA	CATCTCTCATGGCTTCCAGGG	137
Erk2	Human	ACCAGACCTACTGCCAGAGA	ATTTGCTCGATGGTTGGTGC	112
GAPDH	Human	AAGAAGGTGGTGAAGCAGGCG	ACCAGGAAATGAGCTTGACAA	166
SINE	Murine	GCCTTTAATCCCAGCACTTG	CTCTGTGTAGCCCTGGCTGT	103
LINE	Rat	ACTCAGGGGACAACAGATGC	TAGCTGGATCCTCAGGCAGT	150
